# Supplementary material for: Detection of Feline Coronavirus Variants in Cats without Feline Infectious Peritonitis
Source: Viruses. 2022 Jul 29;14(8):1671. doi: 10.3390/v14081671 (PMC9412601; doi:10.3390/v14081671)
Supplement: Supplementary file 1 [file viruses-14-01671-s001.zip › viruses-1729100-supplementary.pdf]

Table S1. Cats without feline infectious peritonitis (FIP) included in the study.

Cat	Breed	Sex	Age	Pathological findings
1	Domestic Shorthair	Female neutered	16 years	Myocarditis, endocardial fibrosis, interstitial pneumonia
2	British Shorthair	Male neutered	13 years	HCM, thrombosis of multiple vessels, end stage CKD
3	Maine Coon	Male neutered	1 year	Internal hydrocephalus, aqueduct stenosis
4	Birman	Female neutered	14 years	Lymphoma of larynx, trachea, and bone marrow
5	Domestic Shorthair	Male neutered	Adult (exact age unknown)	Parvovirosis
6	Domestic Shorthair	Male neutered	18 years	Mast cell tumor in liver, spleen, and intestines
7	Domestic Shorthair	Female neutered	13 years	Sepsis originating from purulent necrotizing enteritis
8	Domestic Shorthair	Male neutered	13 years	Pulmonary adenocarcinoma, thyroid adenoma, CKD
9	Domestic Shorthair	Male neutered	Adult	Parvovirosis and *Taenia* spp. infestation
10	Domestic Shorthair	Male neutered	8 years	Lymphoma in lung, liver, kidney, spleen, and mediastinum
11	Domestic Shorthair	Female neutered	13 years	Multicentric lymphoma
12	Domestic Shorthair	Male neutered	14 years	Soft tissue sarcoma of thymus, spleen, and caecum
13	Domestic Shorthair	Female neutered	6 years	CKD, chronic pyelonephritis
14	Domestic Shorthair	Female	5 months	Purulent pneumonia
15	Domestic Shorthair	Male neutered	Adult (exact age unknown)	HCM, aortic thrombosis
16	Domestic Shorthair	Male neutered	4 years	HCM, pulmonary edema
17	Sphinx	Female neutered	5 years	Purulent necrotizing pneumonia
18	Domestic Shorthair	Male neutered	Adult	Multicentric lymphoma (liver, spleen, brain)

19	Domestic Shorthair	Male neutered	4 years	Oxalate nephrosis, acute kidney injury
20	Domestic Shorthair	Female neutered	9 months	Diaphragmatic herniation
21	Domestic Shorthair	Male neutered	4 years	DCM, pulmonary edema
22	Domestic Shorthair	Male neutered	Adult (exact age unknown)	Blunt trauma with crushing, fragmentation and hemorrhage of the skull
23	Domestic Shorthair	Male neutered	10 years	Lymphoma in kidneys, intestine, and omentum
24	Domestic Shorthair	Female neutered	11 years	Fibrosis of the mitral valve, chronic eosinophilic bronchopneumonia
25	Domestic Shorthair	Male neutered	9 years	Blunt trauma with rib fracture and exit of the heart
26	Domestic Shorthair	Female neutered	15 years	Bronchoalveolar carcinoma
27	Domestic Shorthair	Female neutered	10 years	Lymphoma in intestine, pancreas, mesenteric lymph nodes, kidneys, and omentum
28	Domestic Shorthair	Female neutered	16 years	Hepatic cystadenoma, peritonitis, perihepatitis
29	Domestic Shorthair	Male neutered	8 years	Pneumonia
30	Domestic Shorthair	Male neutered	Adult (exact age unknown)	Purulent bronchopneumonia and interstitial pneumonia
31	Domestic Shorthair	Female neutered	18 years	Thrombosis of the pulmonary vessels, necrotizing hepatitis
32	Maine Coon	Female neutered	13 years	Parvovirosis, intussusception
33	Domestic Shorthair	Male neutered	6 months	Hepatic necrosis and associated bleeding tendency
34	Domestic Shorthair	Female neutered	12 years	HCM, pulmonary edema
35	Domestic Shorthair	Male neutered	14 years	Brain tumor, endomyocardial fibrosis
36	Domestic Shorthair	Male neutered	11 years	RCM, secondary pulmonary edema
37	Domestic Shorthair	Female neutered	Adult (exact age unknown)	Myocarditis, pulmonary edema

38	Domestic Shorthair	Male neutered	16 years	Ruptured hepatocellular adenoma, interstitial pneumonia, CKD
39	Not documented	Not documented	Adult (exact age unknown)	Multimorbidity
40	Domestic Shorthair	Female neutered	13 years	Urolithiasis, end stage CKD
41	Domestic Shorthair	Male neutered	16 years	Thyroid adenoma, secondary changes in the liver (vacuolar degeneration, hepatocellular necrosis), kidneys (interstitial lymphoplasmacytic infiltration), and heart (interstitial fibrosis)
42	Domestic Shorthair	Male neutered	10 years	Adenocarcinoma of the lung with metastasis to various organs
43	Domestic Shorthair	Female neutered	9 years	Thrombosis of numerous pulmonary vessels
44	Domestic Shorthair	Male neutered	8 years	HCM
45	Domestic Shorthair	Male neutered	5 years	Verminous pneumonia, chronic enteritis, chronic interstitial nephritis
46	Domestic Shorthair	Male neutered	12 years	Multicentric lymphoma
47	Domestic Shorthair	Female neutered	7 years	Hepatic lipidosis
48	Domestic Shorthair	Male neutered	16 years	Adenocarcinoma of lung and pleura
49	Domestic Shorthair	Female neutered	17 years	Chronic hepatitis and cholangitis, interstitial pneumonia, CKD
50	Domestic Shorthair	Female neutered	Adult (exact age unknown)	HCM, aortic thrombosis
51	Domestic Shorthair	Female neutered	12 years	Trauma with extensive bleeding
52	British Shorthair	Male neutered	10 years	HCM, pulmonary edema
53	Domestic Shorthair	Male neutered	8 years	Myocarditis, pleuropneumonia
54	Domestic Shorthair	Female neutered	14 years	Pulmonary adenocarcinoma
55	British Shorthair	Female	6 weeks	Volvolus
56	Domestic Shorthair	Male neutered	11 years	RCM, interstitial pneumonia

57	Domestic Shorthair	Male neutered	7 months	*Cryptosporidium* spp. infestation, multifocal necrotizing enterocolitis
58	Domestic Shorthair	Male neutered	4 years	HCM, pulmonary fibrosis, *Taenia* spp. infestation
59	Domestic Shorthair	Female neutered	9 years	Diphteroid necrotizing enteritis, hepatic lipidosis
60	Domestic Shorthair	Male neutered	15 years	RCM, pulmonary edema
61	Domestic Shorthair	Male neutered	12 years	Lymphoma oof liver, kidneys, and gastrointestinal tract
62	Domestic Shorthair	Female neutered	22 years	HCM, pulmonary edema
63	Domestic Shorthair	Female neutered	12 years	HCM, pulmonary edema
64	Domestic Shorthair	Male neutered	20 years	Meningioma
65	Domestic Shorthair	Female neutered	13 years	Multicentric lymphoma
66	Domestic Shorthair	Female neutered	12 years	Embolic purulent nephritis
67	Domestic Shorthair	Male neutered	15 years	Adenocarcinoma of the stomach
68	Birman Mix	Female	1 year	Trauma with multiple hemorrhages
69	Domestic Shorthair	Male neutered	9 years	Chronic fibrosing myocarditis and concentric cardiomyopathy
70	Domestic Shorthair	Male neutered	Adult (exact age unknown)	Hemorrhagic necrotizing cystitis, chronic pyelonephritis, CKD
71	Norwegian Forest Cat	Female neutered	3 years	RCM, interstitial pneumonia
72	Domestic Shorthair	Female neutered	18 years	Hepatocellular adenoma, myocardial fibrosis, CKD
73	Maine Coon	Male neutered	7 years	HCM, pulmonary edema
74	Domestic Shorthair	Female neutered	12 years	Hydronephrosis
75	Domestic Shorthair	Male neutered	10 years	HCM, pulmonary edema, hemorrhagic necrotizing cystitis, CKD
76	Domestic Shorthair	Male neutered	8 years	Enteritis, purulent granulomatous

				tonsillitis and laryngopharyngitis
77	Domestic Shorthair	Male neutered	4 years	Decompensated eccentric left ventricular hypertrophy, pulmonary edema
78	Domestic Shorthair	Female neutered	20 years	Chronic enteritis
79	Domestic Shorthair	Female neutered	6 years	Meningioma
80	Domestic Shorthair	Male neutered	10 years	Generalized lymphoma, hemorrhagic cholecystitis and lymphoplasmacytic cholangiohepatitis
81	Domestic Shorthair	Male neutered	16 years	Transverse fracture of the spine
82	Domestic Shorthair	Male neutered	18 years	Multicentric lymphoma
83	Domestic Shorthair	Female neutered	Adult (exact age unknown)	Hepatolipidosis, CKD, pancreatic islet amyloidosis
84	Domestic Shorthair	Male	3 months	Chronic interstitial pneumonia, pulmonary edema
85	Maine Coon Mix	Male	9 months	Trauma with serial rib fractures, laceration of abdominal wall, hemorrhage into body cavities
86	Domestic Shorthair	Male neutered	9 years	HCM, pulmonary edema
87	British Shorthair	Female neutered	7 years	Hippocampal sclerosis

CKD = chronic kidney disease; DCM = dilated cardiomyopathy; HCM = hypertrophic cardiomyopathy; RCM = restrictive cardiomyopathy.
